# 7^th^ Brazilian Guideline of Arterial Hypertension: Presentation

**DOI:** 10.5935/abc.20160140

**Published:** 2016-09

**Authors:** MVB Malachias

**Table:** Declaration of potential conflict of interest of authors/collaborators of 7th Brazilian Guideline of Arterial Hypertension If the last three years the author/developer of the Guidelines:

Names Membersof the Policy	Participated in clinical studies and / or experimental trials supported by pharmaceutical or equipment related to the guideline in question	Has spoken at events or activities sponsored by industry related to the guideline in question	It was (is) advisory board member or director of a pharmaceutical or equipment	Committees participated in completion of research sponsored by industry	Personal or institutional aid received from industry	Produced scientific papers in journals sponsored by industry	It shares theindustry
Alexandre Alessi	No	No	No	No	No	No	No
Ana Cristina Simões e Silva	No	No	No	No	No	No	No
Andrea Araujo Brandão	Novartis	Biolab, Daiichi Sankyo, Novartis, Servier	No	No	Biolab, Daiichi Sankyo, Novartis, Servier	Biolab, Novartis, Servier, SEM	No
Angela Maria Geraldo Pierin	No	No	No	No	No	No	No
Antonio Carlos Cordeiro Júnior	No	No	No	No	No	No	No
Armando da Rocha Nogueira	No	No	No	No	No	No	No
Audes Diógenes Feitosa	No	No	No	No	No	No	No
Carlos Alberto Machado	No	No	No	No	No	No	No
Carlos Eduardo Poli de Figueiredo	No	No	No	No	No	No	No
Celso Amodeo	Servier	No	Biolab	Servier	Ache, Servier, Merck Serona, AstraZeneca, Biolab	Novartis	No
Cibele Isaac Saad Rodrigues	No	No	No	No	No	No	No
Cláudia Lúcia de Moraes Forjaz	No	No	No	No	No	No	No
Fernando Antonio Almeida	No	No	No	No	No	No	No
Frida Liane Plavnik	No	No	No	No	No	No	No
Paulo César Veiga Jardim	No	Biolab - Servier - Novartis - Aché	No	No	Servier, Ministério da Ciência, CNPq	No	No
Deborah Malta	No	No	No	No	No	No	No
Décio Mion Júnior	No	No	No	No	No	No	No
Dilma do Socorro Moraes de Souza	No	No	No	No	No	No	No
Eduardo Barbosa Coelho	No	No	No	No	No	No	No
Eduardo Costa Duarte Barbosa	Novartis, Servier, MSD, Takeda, Amgen, AstraZeneca, Pfizer	No	No	No	Servier, Biolab	No	No
Elizabeth Muxfeldt	No	No	No	No	No	No	No
Emilton Lima Júnior	No	Biolab	No	No	No	No	No
Fernanda Consolim Colombo	Ache,Novartis	Daiichi Sankyo,Servier,Merck,Boehringer, Novartis	No	No	No	Libbs,Merck	No
Fernando Nobre	No	Novartis Biociências,Merck Soreno,SEM,Sanofi-Aventi,Libbs Farmacêutica	No	Novartis Biociências,Libbs Farmacêutica	Sanofi-Aventis,Libbs Farmacêutica	SEM,Novartis Biociências,Biolab,Torrent,Bayer,Libbs Farmacêutica	No
Flávio Antonio de Oliveira Borelli	No	Servier	No	No	No	No	No
Gil Fernando Salles	No	No	No	No	No	No	No
Gilson Soares Feitosa	No	No	No	No	No	No	No
Giovanio Vieira da Silva	No	No	No	No	No	No	No
Guido Bernardo Aranha Rosito	No	No	No	No	No	No	No
Heitor Moreno Júnior	No	No	No	No	No	No	No
Heno Ferreira Lopes	No	No	No	No	No	No	No
Isabel Cristina Britto Guimarães	No	No	No	No	No	No	No
Ivan Carlos Antonello	No	No	No	No	No	No	No
José Fernando Vilela Martim	No	No	No	No	No	No	No
José Marcio Ribeiro	No	Biolab,B Ingelheim,Pfizer	No	No	Servier	No	No
Juan Yugar Toledo	No	No	No	No	No	No	No
Leda Aparecida Daud Lotaif	No	No	No	No	No	No	No
Lilian Soares da Costa	No	No	No	No	No	No	No
Lucélia Batista Neves Cunha Magalhaes	No	No	No	No	No	No	No
Luciano Ferreira Drager	No	No	No	No	No	No	No
Luis Cuadrado Martins	No	No	No	No	No	No	No
Luiz Aparecido Bortolotto	Novartis	Servier, Biolab, Novartis	No	No	No	Biolab, Novartis, Servier, Baldacci	No
Luiz Carlos Bodanese	Sanofi, GSK, Roche	Sanofi, Novartis, Boehringer	No	Servier, Sanofi, ANGEM	Safoni, ANGEM, GSK	Pfizer	No
Luiz Cesar Nazario Scala	No	No	No	No	No	No	No
Marcia Maria Godoy Gowdak	No	No	No	No	No	No	No
Marcia Regina Simas Torres Klein	No	No	No	No	No	No	No
Marco Antonio Mota Gomes	Novartis, AstraZeneca, Biolab, Daiichi Sankyo,Takeda, Torrent, Libbs, Pfizer, Janssen, Cardios, Omron	No	No	No	No	No	No
Marcus Vinícius Bolívar Malachias	No	Biolab, Chiesi, Medley-Sanofi, Novartis, Pfizer	No	No	No	Biolab, Boehringer-Ingelheim, Novartis	No
Maria Eliane Magalhães	AstraZeneca, Novartis, Sanofi-Aventis, Daiichi Sankyo	AstraZeneca, Pfizer, Sanofi-Aventis, MSD, Chiesi	No	No	AstraZeneca, Pfizer, MSD, Novartis	No	No
Maria Regina Torloni	No	No	No	No	No	No	No
Maria Rita de Figueiredo Lemos Bortolotto	No	No	No	No	No	No	No
Mário Fritsch Toros Neves	Novartis, Servier, AstraZeneca, Pfizer, Sanofi, Libbs	No	Novartis, Servier	No	No	No	No
Miguel Gus	No	No	No	No	No	No	No
Nelson Sass	No	No	No	No	No	No	No
Osni Moreira Filho	No	No	No	No	Biolab, Pfizer, Novartis	EMS	No
Oswaldo Passarelli Júnior	Biolab, Novartis, Daiichi Sankyo						
Paulo Koch Nogueira	No	No	No	No	No	No	No
Roberto Dischinger Miranda	Aché, Astra Zeneca, Bayer, Biolab, Boeringher Ingelheim, MSD, Novartis, Pfizer	No	Bayer, Novartis, Pfizer	No	No	No	No
Roberto Jorge da Silva Franco	No	No	No	No	No	No	No
Rogério Baumgratz de Paula	No	No	No	No	No	No	No
Rui Manuel dos Santos Póvoa	No	No	No	No	No	No	No
Sandra Fuchs	No	No	No	No	No	No	No
Sebastião Ferreira Filho	No	No	No	No	No	No	No
Sergio Kaiser	Bristol-Myers-Squibb, Novaquimica, Abbott	No	No	No	No	No	No
Thiago de Souza Veiga Jardim	Novartis, Astra Zeneca	Biolab, Daiichi Sankyo, Novartis, Astra Zeneca, Servier			Daiichi Sankyo, Servier	Daiichi Sankyo	
Vera Koch	No	No	No	No	No	No	No
Victor Matsudo	No	No	No	Instituto Actigraph	No	No	No
Weimar Kunz Sebba Barroso de Souza	Amgen, AstraZeneca, Bayer, Boehringer, Jonhson & Johnson, Libbs, Lilly, Merck Sharp Dhome, Novartis, Pfizer, Roche, Sanofi Aventis, Daiichi Sankyo, Torrent	AstraZeneca, Biolab, Boehringer, Lilly, Servier, Torrent	No	No	No	No	No
Wille Oigman Cobrei	Libbs	No	No	No	No	No	No

## Introduction

On behalf of the Brazilian Society of Cardiology, Brazilian Society of Hypertension and Brazilian Society of Nephrology, I present to you the 7^th^ Brazilian Guideline of Arterial Hypertension, result of the joint work of an expert team of renowned professionals appointed by their respective medical societies.

The production of this new guideline was necessary to update the knowledge accumulated over the past years, considering the Brazilian reality and clinical practice. The guidance and recommendations contained in this document reflect the evidence of the effectiveness of the interventions. The present text does not deal specifically with cost-effectiveness analyses. The major objective of the medical societies and authors is to guide health professionals on the preventive measures and care for individuals with arterial hypertension, aiming at reducing the complications of arterial hypertension, considered the major risk factor for cardiovascular disease.

The following table was used as reference for the grades of recommendation and levels of evidence.

**Table:** 

Grades of recommendation:
Class I: Conditions to which there is conclusive evidence or at least general consensus concerning the safety and usefulness/efficacy of the intervention;
Class II: Conditions to which there is conflicting evidence and/or disagreement of opinion concerning the safety and usefulness/efficacy of the intervention;
Class IIa: Evidence/opinion in favor of the intervention. Most experts agree;
Class IIb: Less well-established safety and usefulness/efficacy, without predominance of favorable opinions on the intervention;
Class III: Conditions to which there is evidence and/or consensus that the intervention is not useful/effective, and, in some cases, even harmful.

Levels of evidence:
Level A: Data derived from multiple consistent randomized controlled clinical trials, and/or a robust meta-analysis of randomized clinical trials;
Level B: Data derived from a less robust meta-analysis, one single randomized trial or non- randomized trials (observational);
Level C: Data derived from consensual expert opinion.
We hope the concepts expressed in this guideline can be widely spread to a better and more comprehensive care of individuals with arterial hypertension.

Marcus Vinícius Bolívar Malachias

**Table:** Abbreviations of the Brazilian Guideline of AH

ABI	Ankle-brachial index	ICU	Intensive care unit
ABPM	Ambulatory blood pressure monitoring	IDF	International Diabetes Federation
AC	Abdominal circumference	IGF-1	Insulin-like growth factor 1
ACC	American College of Cardiology	IHD	Ischemic heart diseases
ACEI	Angiotensin-converting-enzyme inhibitor	IMT	Intima-media thickness
ACTH	Adrenocorticotropin	IPEMs	State Departments of Weights and Measures
AH	Arterial hypertension	ISH	Isolated systolic hypertension
AHA	American Heart Association	IV	Intravenous
AHI	Apnea-hypopnea index	LVH	Left ventricular hypertrophy
AKI	Acute kidney injury	LVMI	Left ventricular mass index
Aldo	Aldosterone	MH	Masked hypertension
AMI	Acute myocardial infarction	MI	Myocardial infarction
APA	Aldosterone-producing adenoma	MIBG	Metaiodobenzylguanidine
APE	Acute pulmonary edema	MRI	Magnetic resonance imaging
ARB	Angiotensin II receptor blocker	MS	Metabolic syndrome
BB	Beta-blocker	NKF	National Kidney Foundation
BMI	Body mass index	NPT	Non-pharmacological treatment
BP	Blood pressure	OSAHS	*Obstructive sleep apnea-hypopnea syndrome*
CAD	Coronary artery disease	PAD	Peripheral arterial disease
CAH	Chronic arterial hypertension	PE	Preeclampsia
CbVD	Cerebrovascular diseases	PH	Prehypertension
CCB	Calcium-channel blocker	PHA	Primary hyperaldosteronism
CDC	Centers for Disease Control and Prevention	PHEO	Pheochromocytoma
CHF	Congestive heart failure	PNS	Brazilian National Health Survey
CKD	Chronic kidney disease	POF	Survey on Family Income
COPD	Chronic obstructive pulmonary disease	PP	Pulse pressure
CPAP	Continuous positive airway pressure	PRA	Plasma renin activity
CrCl	Creatinine clearance	PSNS	Parasympathetic nervous system
CS	Cushing’s syndrome	PTH	Parathormone
CT	Computed tomography	PVR	Peripheral vascular resistance
CV	Cardiovascular	PWV	Pulse wave velocity
CVD	Cardiovascular disease	RAAS	Renin-angiotensin-aldosterone system
CVRF	Cardiovascular risk factor	RAH	Resistant arterial hypertension
DAH	Diastolic arterial hypertension	RBMLQ	Brazilian Legal Metrology and Quality Network
DBP	Diastolic blood pressure	RF	Risk factor
DHA	Docosahexaenoic acid	RVAH	Renovascular arterial hypertension
DIU	Diuretic	SAH	Systemic arterial hypertension
DM	Diabetes mellitus	SBP	Systolic blood pressure
EPA	Eicosapentaenoic acid	SNP	Sodium nitroprusside
GFR	Glomerular filtration rate	SNS	Sympathetic nervous system
GH	Growth hormone	SUS	Brazilian Unified Health Care System
GRS	Global Risk Score	TC	Total cholesterol
HbA1c	Glycated hemoglobin	TIA	Transient ischemic attack
HBPM	Home blood pressure monitoring	TOD	Target-organ damage
HC	Hypertensive crisis	TSH	Thyroid stimulating hormone
HD	Hypertensive diseases	UACR	Urine albumin/creatinine ratio
HDL-C	High-density lipoprotein cholesterol	US	Ultrasonography
HE	Hypertensive emergency	VIGITEL	Surveillance for Risk Factors and Protection Against Chronic Diseases via Telephone Inquiry
HF	Heart failure	WCE	White-coat effect
HR	Heart rate	WCH	White-coat hypertension
HU	Hypertensive urgency		

**Marcus Vinícius Bolívar
Malachias**

